# Service Characteristics and Geographical Variation in Compulsory Hospitalisation: An Exploratory Random Effects Within–Between Analysis of Norwegian Municipalities, 2015–2018

**DOI:** 10.3389/fpsyt.2021.737698

**Published:** 2021-12-09

**Authors:** Tore Hofstad, Jorun Rugkåsa, Solveig Osborg Ose, Olav Nyttingnes, Solveig Helene Høymork Kjus, Tonje Lossius Husum

**Affiliations:** ^1^Centre for Medical Ethics, Institute of Health and Society, University of Oslo, Oslo, Norway; ^2^Health Services Research Unit, Akershus University Hospital, Lørenskog, Norway; ^3^Centre for Care Research, University of South-Eastern Norway, Porsgrunn, Norway; ^4^SINTEF, Health Services Research, Trondheim, Norway; ^5^NTNU Social Research, Norwegian Resource Centre for Community Mental Health, Trondheim, Norway; ^6^Department of Nursing and Health Promotion, Faculty of Health Sciences, Oslo Metropolitan University, Oslo, Norway

**Keywords:** compulsory hospitalisation, geographical variation, service characteristics, nested generalised linear mixed model, random effects within-between models

## Abstract

**Background:** Compulsory hospitalisation in mental healthcare is contested. For ethical and legal reasons, it should only be used as a last resort. Geographical variation could indicate that some areas employ compulsory hospitalisation more frequently than is strictly necessary. Explaining variation in compulsory hospitalisation might contribute to reducing overuse, but research on associations with service characteristics remains patchy.

**Objectives:** We aimed to investigate the associations between the levels of compulsory hospitalisation and the characteristics of primary mental health services in Norway between 2015 and 2018 and the amount of variance explained by groups of explanatory variables.

**Methods:** We applied random-effects within–between Poisson regression of 461 municipalities/city districts, nested within 72 community mental health centre catchment areas (*N* = 1,828 municipality-years).

**Results:** More general practitioners, mental health nurses, and the total labour-years in municipal mental health and addiction services per population are associated with lower levels of compulsory hospitalisations within the same areas, as measured by both persons (inpatients) and events (hospitalisations). Areas that, on average, have more general practitioners and public housing per population have lower levels of compulsory hospitalisation, while higher levels of compulsory hospitalisation are seen in areas with a longer history of supported employment and the systematic gathering of service users' experiences. In combination, all the variables, including the control variables, could account for 39–40% of the variation, with 5–6% related to municipal health services.

**Conclusion:** Strengthening primary mental healthcare by increasing the number of general practitioners and mental health workers can reduce the use of compulsory hospitalisation and improve the quality of health services.

## Introduction

Compulsory hospitalisation deprives patients of their liberty and remains contested. This is due to both negative experiences with coercion reported by patients (1, 2) and a lack of reliable studies that demonstrate beneficial outcomes of such hospitalisations. Compulsory hospitalisation is bound by law as a last resort, after voluntary care has been tried or deemed futile. There have been multiple initiatives to reduce its use, including the European Council's recommendation to abolish coercion in mental healthcare (3). Yet, clinicians continue to assess patients to occasionally require admission against their will (4), for instance to prevent serious harm, which might partly explain why no jurisdiction seems able to do entirely away with coercive practice (5).

The observed variation in the levels of compulsory hospitalisation within jurisdictions is noteworthy (6–12) and shows up to a sixfold difference between the highest and lowest average rate of compulsory hospitalisation per 100,000 inhabitants in hospital catchment areas (13). If such variation does not have any clear explanation, this could indicate that certain areas use more compulsion than strictly necessary and, thus, have a potential for reduction.

The risk of compulsory hospitalisation has repeatedly been linked to individual level characteristics, such as the presence of severe mental illness (SMI), previous compulsory hospitalisation, male gender, single or divorced marital status, unemployment, and receipt of welfare benefits (14). However, only a few studies have focused on the organisation of health services, which might complement our understanding of what we consider risk or preventive factors, for compulsory hospitalisation.

There are reasons to believe that the existence of supportive, voluntary alternatives acceptable to both patients and health professionals can reduce the need for compulsory hospitalisation by facilitating recovery or crisis management. A study from Belgium showed that the lack of less restrictive alternatives was a stronger predictor for compulsory hospitalisation than was the presence of a mental disorder or dangerousness (15). This suggests that, to help minimise excessive compulsion usage, it is important to ascertain whether differences in the organisation and resources of primary mental health services are associated with more, or less, compulsory hospitalisation (16). To widen the scope of the existing literature, which primarily focuses on patient-related factors, we will specifically investigate the role of service characteristics, and we select factors that have been associated with compulsory admissions in the literature or, there is good reason to believe have such associations.

### Service Characteristics Related to Compulsory Hospitalisation

Compulsory hospitalisation has been associated with the size and constitution of the primary mental health *labour force*. A report from Norway found lower rates of compulsory hospitalisation in hospital catchment areas with more labour-years in primary mental health services per population (17). Poor housing or homelessness can both be a consequence of and a risk factor for SMI. Providing *public housing* for individuals in high-risk groups might thus reduce the need for compulsory hospitalisation. A French study found lower rates of involuntary inpatients in areas with increased housing capacity for disabled individuals and slightly higher rates in areas with more general practitioners (GPs) (6). Explanations for the latter finding ranged from GPs' lack of ability to identify and treat mental health needs to a possible confounding with urbanisation.

Unemployment has been associated with a higher risk of compulsory hospitalisation (18). Unemployment reduces income and impacts social status, both of which might lead to the deterioration of mental well-being. In addition, unemployment might also result from mental illness. Area-based coordinated initiatives for *employment support* that aim to facilitate the employment of individuals with SMI (19) might therefore impact the risk of compulsory hospitalisation.

It seems likely that the quality of *cooperation between service levels* might impact the levels of compulsory hospitalisation. In Norway, staff in specialist services are expected to supervise and offer consultations to their colleagues in primary health services (20). Those working in these services have identified good collaboration as a factor that has the potential to reduce the use of compulsion (21). Furthermore, *recovery-oriented practice* focuses on rehabilitation and empowerment (22). Recovery principles, including the *systematic gathering of experiences from service users*, can influence how primary mental health services are organised and delivered, for instance by contributing unique expertise through lived experience (23), which might increase the likelihood of services being received voluntarily.

Finally, *early intervention services* that seek to identify mental health problems and intervene at an early stage (24) could theoretically be associated with the level of compulsory hospitalisation.

### Aims

The overarching aim of this investigation is to explore the relationships between the levels of compulsory hospitalisation and the organisation of primary mental health services. We will answer the following research questions:

1) What is the direction and the strength of association between selected characteristics of primary mental health services and the area level of compulsory hospitalisation?
2) How much of the variation in compulsory hospitalisation is accounted for by the area's *age distribution, deprivation level, SMI prevalence*, and *municipal mental health services?*

## Methods

### Study Design

The study design is a retrospective exploratory panel analysis with hierarchical models that account for clusters at different levels, using an approach that separates variation within and between areas.

### Study Context

Primary healthcare in Norway is delivered by local authorities, which are also responsible for social care and public housing. These consist of municipalities and the city districts of the four largest cities (Oslo, Stavanger, Bergen, and Trondheim). These 461 areas (hereafter referred to as municipalities) constitute our level of analysis. All use of compulsory mental healthcare is initiated by specialist services, which is delivered by 22 state-owned Hospital Trusts. The Hospital Trusts have acute inpatient wards and Community Mental Health Centres (CMHC) that deliver decentralised specialist treatment, often in cooperation with the municipalities. During the study period, the number of municipalities reduced from 459 to 457 in 2017 and to 453 in 2018, and the CMHC catchment areas reduced from 69 to 67 in 2017 and to 65 in 2018.

Compulsory hospitalisation is regulated by the 1999 Norwegian Mental Health Care Act. The main legal criterion for admitting patients for involuntary observation or treatment is that the patient must suffer from a serious mental disorder. Additionally, voluntariness must have been tried, the patient's condition must be likely to deteriorate without treatment, or the patient poses an immediate risk to themselves or others. From 2017, compulsory care is only permitted for patients who lack the capacity to consent to treatment, unless there is immediate and serious risk to the patient's own life or the life or health of others.

### Sample and Data Sources

Individual level data on all contacts with specialist services in Norway are routinely recorded in the National Patient Register (25). We acquired data for each episode of compulsory hospitalisation during 2015–2018 and for each contact with specialist services by people with SMI. The population at risk of compulsory hospitalisation was defined as all individuals between 18 and 65 years residing within a Norwegian municipality during the study period. This range was chosen since services are organised differently for the other age groups. We excluded individuals without a Norwegian identification number or those from whom information on residency was missing (1 and <0.001% of people compulsorily hospitalised, respectively).

Information on population, public housing, and labour-years of GPs and mental health nurses was collected from Statistics Norway. To calculate the population-based rates, we included all individuals between 18 and 65 years residing in each municipality during the study period.

Information on the remaining service characteristics was obtained from the annual report to the Norwegian Directorate of Health by the mental health and addiction services in each municipality (26). Unemployment data were provided by the Norwegian Labour and Welfare Administration. Table 1 contains the description and data source for outcomes and the explanatory and control variables. More detailed information about the data sources can be found in the Appendix.

**Table 1:** Description of measures and data sources.

**Name of measure**	**Description of measure**	**Data source**
**Outcomes**		
Compulsory hospitalisations	Number of episodes of compulsory hospitalisation per year. Population aged 18-65	NPR
Compulsory hospitalised patients	Number of individuals hospitalised compulsory per year.	NPR
**Explanatory variables**		
Overall labour-years	Total number of labour-years within municipal mental health and addiction services per 1,000 population.	IS 24/8
General practitioners	Labour-years for physicians in the municipal health and care services per 1,000 population.	Statistics Norway
Mental health nurses	Labour-years for psychiatric nurses in the municipal health and care services per 1,000 population.	Statistics Norway
Public housing	Total number of municipal disposed dwellings per 100 inhabitant.	Statistics Norway
Housing first	Has the municipality/city district employed “Housing First?” (Yes/No).	IS 24/8
Employment support	Has the municipality used IPS/Supported Employment within mental health and substance misuse work? (Yes/No).	IS 24/8
Quality of cooperation	How do you evaluate that the cooperation agreement between municipality and health trust.	IS 24/8
between municipality	is working for adults with mental health difficulties/illness? (Very good/Good/Medium/Poor/Very poor).	
and specialist services		
Early intervention	Has the municipality made initiatives to uncover mental health or addiction problems as early as possible? (Yes/No).	IS 24/8
Recovery	To what extent would you say that the services in mental health and addiction in your municipality is recovery oriented?	IS 24/8
Perspectives	(Very great extent/Great extent/Some extent/Small extent/Very small extent).	
Service users'	Has the municipality in a systematic way gathered user experiences within mental health or addiction services	IS 24/8
Perspectives	during the last 12 months? (Yes/No).	
**Control variables**		
Share of population aged 20–39	Number of individuals aged 20–39 divided by total population in area.	Statistics Norway
Share of population 65 +	Number of individuals older than 65 years divided by total population in area.	Statistics Norway
SMI per 1,000	Annual number of people with severe mental illness who was in contact with specialist services	NPR
	divided by total population in area, multiplied by 1,000.	
Crowded housing	Percentage of households that live in crowded housing.	Statistics Norway
Unemployment rate	Percentage of work force, age 15–74, that is unemployed.	Norwegian Labour and Welfare Administration

### Variables

We have previously shown that the geographical variation in the level of compulsory hospitalisation appears larger when rates are based on the number of hospitalisations (events), rather than the number of patients hospitalised (individuals), and that including both outcomes is likely to yield a more encompassing picture (13). Two outcome measures were therefore employed in the present analysis: (i) the annual number of compulsory hospitalisations (for observation or treatment) and (ii) the annual number of patients with at least one compulsory hospitalisation.

The municipal mental health and addiction services are interdisciplinary, and the total number of labour-years included nurses, healthcare workers, GPs, and psychologists. The rates were calculated by dividing counts by the population aged 18–65 years. The question of the perceived quality of the cooperation between primary and secondary mental health services and the question on the recovery orientation of services were scored by service managers in each municipality. Answers for the latter two were recoded as numeric variables ranging from one to five, where a higher score represented better cooperation or greater extent of recovery orientation. Housing First, employment support, early intervention, and service users' perspectives were included as dummy variables. Data on recovery perspectives were only available for 2017–2018, while data on Housing First and early intervention were only available for 2015.

To adjust for differing risks due to age distribution, the population share aged 20–39 years was included as a covariate since this age group has a higher risk of compulsory hospitalisation. Similarly, the municipality's share of population aged 65 years and over was included due to the lower risk in this age group. The annual number of individuals who had or received a diagnosis of SMI and were in contact with specialist services, divided by the area's at-risk population and multiplied by 1,000, was included to account for differences in case mix. SMI was defined according to the International Classification of Diseases 10th revision (ICD-10) diagnosis codes F20–F31 (27). To account for differences in area deprivation level, the proportion of people living in crowded housing and the unemployment rate were included as covariates. Finally, dummy variables were added for each year. Neither of these control variables were assumed to be caused by the outcomes or the exposures of interest, but they could theoretically impact both.

### Statistical Analysis

In order to answer research question one, associations between the health service characteristics and compulsory hospitalisations were explored using generalised linear mixed models, which account for non-independence of observations (28). Random intercepts for municipalities nested within CMHC catchment areas were modelled to allow for differences in compulsory hospitalisation between areas at both levels. A random-effects within–between approach was employed, as recommended in the literature (29). Between-area associations are investigated by comparing areas cross-sectionally, while longitudinal data also contain within-area variance which can be used to compare each area with itself at different time points. In order to disentangle the two sources of variation, each time-varying predictor was split into two, where the municipality average during the study period was used to estimate between-area associations, while the deviation from the municipality average was used to estimate within-area associations. These within-area associations are useful for predicting change in the levels of compulsory hospitalisation when specific service characteristics change, as they are not biased by omitted variables at the municipality level since all unmeasured time-invariant variables are absorbed into the between effect. For the binary variables, the between association represents the proportion of time the municipality employed that measure.

Since the outcomes were counts, a Poisson error distribution was assumed and a log link function was used (30). Since the municipalities differ in population size, the log of the population aged 18–65 years was used as offset, which changed the outcome to rate per population. Rather than combining all variables in one large model, separate models were run for each explanatory variable to avoid conditioning on potential colliders and mediators. Models were fit using the Laplace approximation. In order to quantify the predicted change in the levels of compulsory hospitalisation between and within areas, conditional effect plots were created for the four continuous explanatory variables. The equations for the hierarchical models and the descriptions of the effect plots are found in the Appendix. For the two explanatory variables where only one wave of data was available, cross-sectional analysis in the form of Poisson regression was performed using CMHC catchment area as fixed effect.

In order to answer the second research question of variance explained for groups of explanatory variables, the marginal *R*^2^ suggested by Nakagawa and Schielzeth was calculated (31), which only considers the variance of the modelled variables, in other words the fixed effects, and not the random effects. Separate values were calculated for *age distribution in the area; area deprivation level*, which included the unemployment rate and share living in crowded housing; and *illness prevalence*, which is the number of individuals diagnosed with SMI who were in contact with specialist services each year. All variables on *service characteristics* were included in the same model in order to evaluate the combined explanatory power. The explained variance of the labour-years of mental health nurses and recovery perspectives was estimated in a separate model due to the higher number of missing values. Finally, all groups were included in the same model in order to estimate the *total* variance explained by all groups of variables. To ensure that the same number of units were compared for all groups of variables, only units without missing values for all groups of variables were included in these analyses. Finally, model performance and robustness were checked by estimating models differing in nesting, models controlling for grand mean change of predictors over time, and models using the fixed-effect Poisson estimator with White's heteroscedasticity robust standard errors and area-clustered standard errors. All analyses were performed using R version 4.0.3 (32) and the following packages: *tidyverse* (33) and *data.table* (34) for data wrangling, *ggplot2* for graphs (35), and *ggeffects* 1.0.2 (36) for calculating marginal effects. For multilevel analyses, *lme4* 1.1.26 (37) was used with the “bobyqa” optimiser. For the fixed-effect Poisson estimator, the *fixest* package was used (38). The *performance* 0.7.0 package (39) was used to evaluate model performance and to calculate *R*^2^.

### Missing Values

Completeness across all data sources was in general very good, except for three explanatory variables with 12–21% missing. Two of these were only available for 2015 (Housing First and early intervention). In these two cases, multiple imputation was performed using the *mice* package (40), with default settings and 20 imputations. For the third variable, labour-years of mental health nurses, 392 observations (21.4%) were missing among municipality-years, and 51 municipalities (12.2%) had missing values for the level 2 between-area association. This and the remaining variables with missing values were handled by listwise deletion.

### Ethics

The South-Eastern Regional Research Ethics Committee gave permission to analyse de-identified registry data, but otherwise deemed the study as falling outside their remit as specified by the Norwegian Health Research Act (ref. 2018/795). The project was therefore approved by the Privacy Ombudsman at Akershus University Hospital following a detailed data protection impact assessment (ref. 2018-090).

## Results

### Descriptive Statistics

The average number of compulsory hospitalisations in each municipality varied from 0 to 206, with a mean value of 14.6. Descriptive statistics of the municipalities' average values during the study period can be seen in Table 2.

**Table 2:** Characteristics of Norwegian municipalities and city districts, 2015–2018.

**Name of measure**	**Municipality-years**	**% missing**	**Mean**	**Min**	**Max**	**SD**	**Distribution**
Compulsory hospitalisations	1,828	0	14.6	0	206	25.2	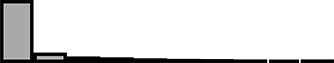
Compulsory hospitalised patients	1,828	0	10.8	0	120	18.0	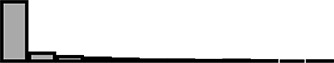
Population aged 18–65	1,828	0	7,198	120	76,681	10,057	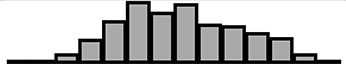 ^*^
Share of population aged 20–39	1,828	0	23.6	16.2	69.5	5.0	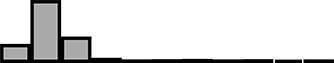
Share of population 65+	1,828	0	18.3	3.0	28.7	4.0	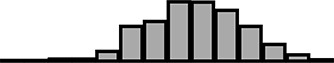
Severe mental illness per 1,000	1,828	0	1.6	0	9.1	0.9	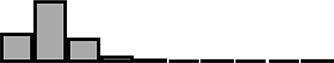
Crowded housing share	1,828	0	8.2	2.4	29.6	3.7	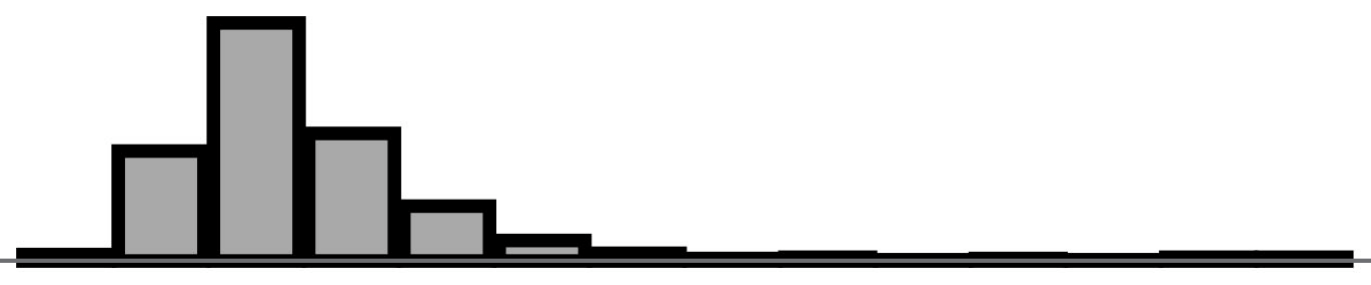
Unemployment share	1,821	0.4	2.3	0.5	9.5	1.1	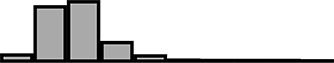
Labour years per 1,000
Total in municipal mental health	1,828	0	3.7	0.7	14	1.6	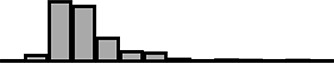
General practitioners	1,828	0	1.3	0.2	3.4	0.5	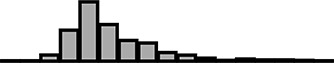
Mental health nurses	1,604	12.2	0.4	0.01	2.3	0.4	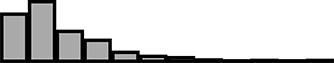
Public housing per 100	1,828	0	2.6	0.1	9.0	1.2	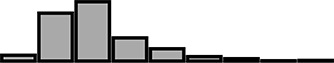
Housing first^†^	387	15.7	0.1	0	1	0.4	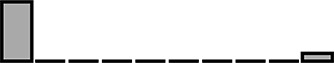
Supported employment	1,824	0.2	0.2	0	1	0.3	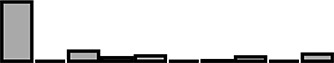
Quality of cooperation	1,820	0.4	3.5	1	5	0.5	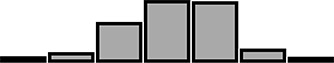
Early intervention^†^	403	12.2	0.8	0	1	0.4	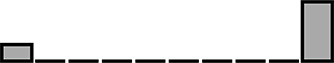
Recovery perspectives^††^	900	1.1	3.8	1	5	0.7	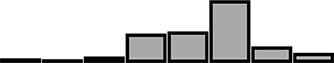
Systematic gathering of user experiences	1,824	0.2	0.5	0	1	0.3	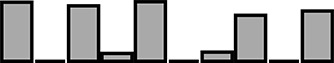

*Descriptive statistics are based on the average values for Norwegian municipality/city districts in 2015-2018*.

* *Distribution shows logged values*.

† *Data available from 2015*.

†† *Data available from 2017 and 2018*.

### Associations Between Features of Mental Health Services and Levels of Compulsory Hospitalisation

The intraclass correlation is equal to the variance partition coefficient for models with only random intercepts and shows the amount of variation due to systematic differences between the municipalities nested within the CMHC catchment areas. For compulsorily hospitalised patients, the clustering accounted for 40% of the variation (CMHC = 15%, municipality = 25%); for compulsory hospitalisations, the clustering accounted for 62% of the variation (CMHC = 19%, municipality = 43%).

**Figure 1:**
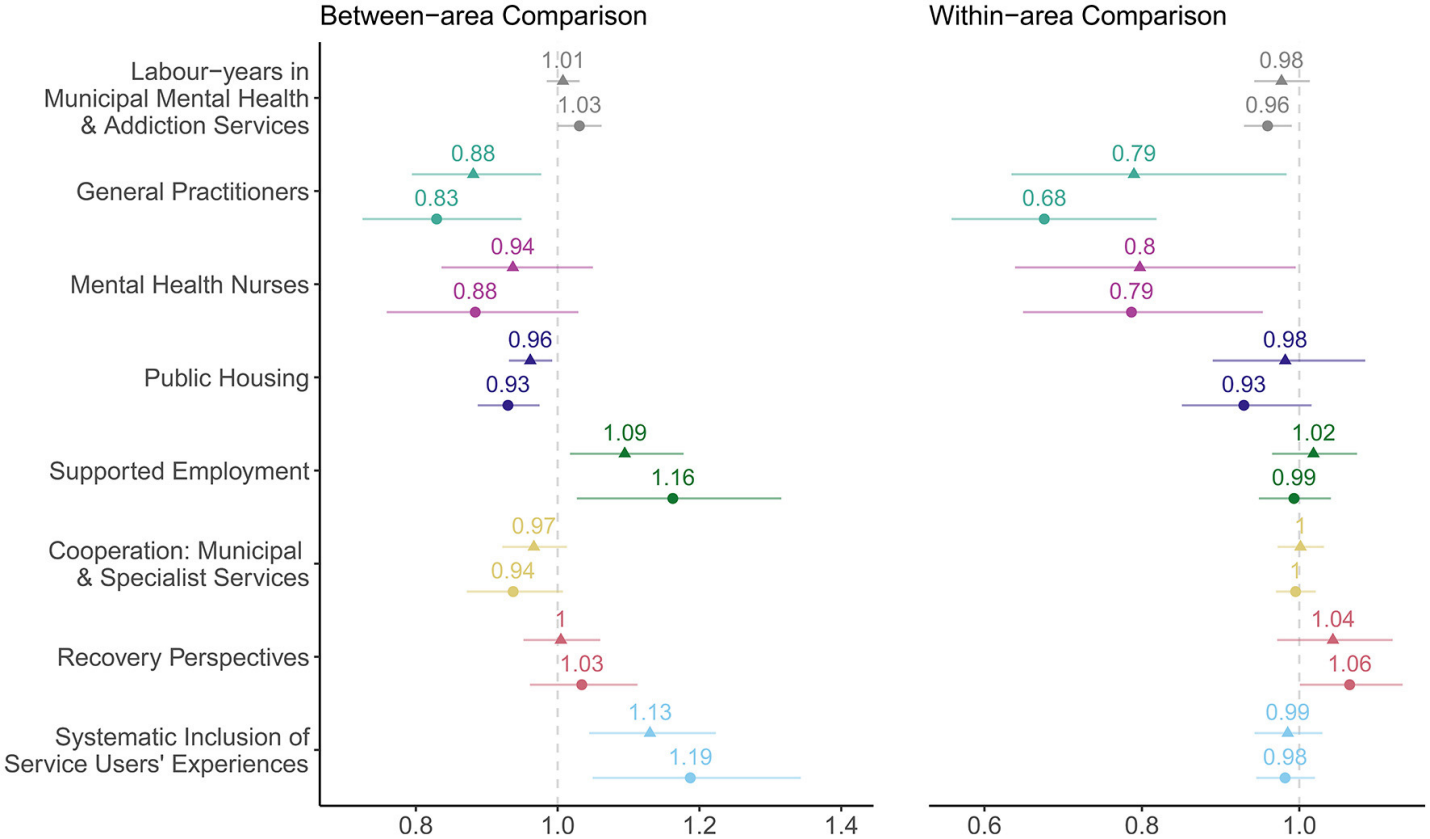
Between- and within-area associations of municipal mental health service in Norway, 2015–2018. ▴, patients; ∙, hospitalisations. Rate ratios with 95% Wald confidence intervals.

Figure 1 shows the exponentiated regression coefficients from eight different multilevel Poisson models, which can be interpreted as rate ratios. They represent the relative change in the annual rates of patients and hospitalisations per population that would be expected for a one unit increase in each explanatory variable, while accounting for control variables. Unadjusted models are included in the Appendix, along with the robustness checks and model performance.

Most associations were larger when the outcome was hospitalisations compared to patients. The largest unstandardised rate ratios were seen within areas for GPs and mental health nurses. A 12–16% increase of GPs per population from the area average was associated with a reduction of one compulsory hospitalisation, as seen in the conditional effect plots in the Appendix. For the total number of labour-years in municipal mental health and addiction services, the trends were weaker, and the association was marginally in the opposite direction for the between-area comparisons.

Higher numbers of public housing were also associated with lower levels of compulsory hospitalisation, as measured by both patients and hospitalisations, and both within and between municipalities. In contrast, areas with supported employment had 0.09 times higher rates of compulsorily hospitalised patients and 0.16 times higher rates of compulsory hospitalisation compared to areas without supported employment.

For the measurements of cooperation between municipal and specialist services, there was no discernible within association, but municipalities that more often rated the cooperation to be good had lower levels of compulsory hospitalisation compared to municipalities where the cooperation was rated as poorer; however, the 95% confidence intervals (CIs) included 1.

For recovery perspectives in municipal services, three of four associations pointed towards higher levels of compulsory hospitalisations, particularly within municipalities. Similarly, areas that systematically gathered service user's perspectives had 0.13 times higher rates of compulsorily hospitalised patients and 0.19 times higher rates of compulsory hospitalisation compared to areas that did not gather service user's perspectives systematically.

The cross-sectional analyses (only reported in text) showed that the levels of compulsory hospitalisation were higher in municipalities that reported initiatives to uncover mental health problems as early as possible, after adjusting for area demographics and socio-economic status (patients: β = 1.1, 95% CI = 0.96–1.26, *p* = 0.16; hospitalisations: β = 1.19, 95% CI = 1.06–1.34, *p* = 0.004) compared to municipalities without such measures. Similarly, municipalities that employed Housing First had more compulsorily hospitalised patients compared to areas without a Housing First policy (patients: β = 1.14, 95% CI = 1.00–1.30, *p* = 0.049; hospitalisations: β = 0.97, 95% CI = 0.82–1.14, *p* = 0.69).

### Amount of Variance Explained by Groups of Explanatory Variables

**Figure 2:**
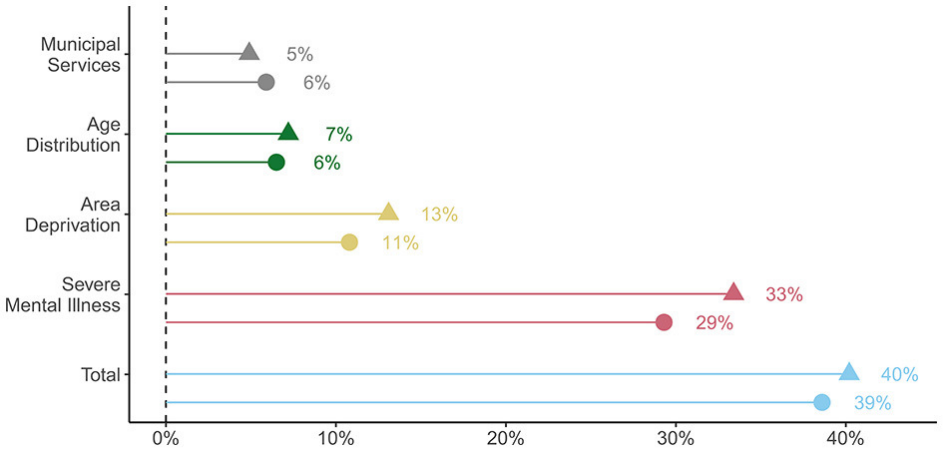
Amount of total variance explained by groups of explanatory variables. ▴, patients; ∙, hospitalisations.

In total, all the groups of variables accounted for 39–40% of the variation, as seen in Figure 2. The municipal mental health services accounted for 5% of the variation in compulsorily hospitalised patients and 6% of compulsory hospitalisations. The separate model containing the labour-years of mental health nurses and recovery perspectives accounted for roughly 1.5% of the variation. In contrast, the annual number of individuals diagnosed with SMI per 1,000 alone accounted for 29–33% of the variation.

## Discussion

This study showed considerable geographical variation in compulsory hospitalisation between Norwegian municipalities in 2015–2018, which was associated with several characteristics of the municipal mental health services, both when comparing areas cross-sectionally and when comparing each area with itself over time. Higher levels of labour-years of GPs and mental health nurses were associated with lower levels of compulsory hospitalisation. Furthermore, areas that on average had more public housing had lower levels of compulsory hospitalisation compared to areas that on average had less public housing. Higher levels of compulsory hospitalisation were observed in areas that had strategies for employment support for longer time periods compared to areas with shorter or no employment support. Similarly, areas that reported to systematically include user experiences in multiple years showed higher levels of compulsory hospitalisation compared to areas that, to a lower extent, included user experiences. Somewhat higher levels of compulsory hospitalisation were also seen within municipalities over time for services reporting high levels of recovery orientation. Combined, the variables on municipal mental health services could account for a modest 5–6% of the observed variation.

Our findings suggest that GPs play an important role in reducing compulsory hospitalisations. GPs often serve as the first, and only, health service for mental disorders and as the gateway to other services. More GP capacity may provide more time for treatment and continuity and help patients avoid deterioration. In a Norwegian study, referrals to compulsory hospitalisation were more often made by physicians who did not know the patient (41), as opposed to GPs. Our results imply that the risk of compulsory hospitalisation could increase in areas that struggle to maintain their GP-to-inhabitant ratio and that strengthening the GP service could aid in preventing compulsory admissions. This association was slightly weaker between areas, but still robust to different model specifications. This is seemingly in contrast to the finding of Gandré et al. (6) from France of increased levels of compulsorily hospitalised patients in areas with more GPs. However, their variable suffered from collinearity, and the 95% CI for the regression coefficient included 1. Furthermore, since the organisation and the content of healthcare services differ between countries, the results are not directly comparable.

The labour-years of mental health nurses showed somewhat weaker negative within- and between-area associations with both compulsory hospitalisations and compulsorily hospitalised patients, which is in line with previous findings from Norway (17) and Finland (42). More labour-years can enable frequent contact and group activities, facilitating peer discussions and the development of a therapeutic alliance (43). Furthermore, increased availability of personalised supervision for coping and maintaining a stable everyday life can enable early discovery and prevent the deterioration of known SMI, reducing the need for compulsory hospitalisation. Conversely, in small, rural municipalities where one or two mental health nurses might represent the only staff, services are more vulnerable, and challenges can arise when there is sick leave among the staff, or when the need for service arises outside of office hours.

The total number of labour-years in municipal mental health services showed less pronounced associations, but demonstrated the benefits of separating within- and between-variation (29). More labour-years within each area was associated with fewer compulsory hospitalisations, while municipalities that on average employed more labour-years had higher levels of compulsory hospitalisation compared to municipalities with fewer labour-years. Such a finding could emerge if more labour-years resulted in reduced levels of compulsory mental healthcare, but that the increase in labour-years primarily occurred in areas with challenging case mix and high rates of compulsory mental healthcare.

There was a slightly lower rate of compulsory hospitalisations for each additional public housing per 100 inhabitants. Insecurity regarding living conditions is likely to have a major impact on individuals who are already vulnerable (21, 44).

There were more compulsorily hospitalised patients in areas that employed Housing First and slightly higher levels of compulsory hospitalisations in areas that had employment support. These are services that are found in a minority of municipalities and are likely to be initiated based on needs. As these services are not mandatory, they will have to be prioritised in competition with other municipal initiatives. Consequently, employment support or Housing First does not necessarily increase the risk of compulsory hospitalisation, but could rather indicate that these programs may have been initiated in areas with more compulsory hospitalisation.

Concerning cooperation between municipalities and health trusts, we observed slightly lower levels of compulsory hospitalisation in municipalities that gave a favourable rating of their cooperation with specialist services compared to areas with a less favourable rating. This is in line with perspectives from professionals within primary mental health services, who considered poor collaboration with secondary mental health services a risk factor for compulsory hospitalisation (21).

Municipalities that reported initiatives to uncover mental health problems as early as possible showed moderately higher levels of compulsory hospitalisation. One explanation could be that these initiatives uncover individuals who are in need of treatment, but are unable or unwilling to receive voluntary treatment, in line with the findings of Weich et al. (45). Their study identified higher awareness of treatment needs as a possible explanation for the higher levels of compulsory hospitalisation. Alternatively, it could be that areas with low levels of compulsory hospitalisation see less need to initiate early intervention measures.

Recovery-oriented services showed a somewhat surprising positive, but weak, within-area association. Since we only had access to 2 years of data for this variable, the within-area comparisons are less likely to reliably measure weak associations. Furthermore, our measure says nothing about what a recovery-oriented service actually implies (46). Still, we remain open to the possibility that applying more recovery perspectives in municipal mental health services could result in more compulsory hospitalisations, and that recovery perspectives may also exist within services with high levels of compulsory hospitalisation (47).

The systematic gathering of user experiences was associated with slightly higher levels of compulsory hospitalisations between areas. A possible explanation of this could be that municipalities with higher levels of compulsory hospitalisation are more inclined to include user experiences. However, the users of municipal mental health services who inform the municipalities might not be the patient group most likely to be compulsorily hospitalised, which would give less reason to expect reductive effects of including user experiences.

In summary, several of the explanatory variables showed negative associations with the levels of compulsory hospitalisation. Meanwhile, some measures, such as Housing First, employment support, and inclusion of user perspectives, showed somewhat surprising between-area associations. This raises the question whether these measures were initiated based on needs, or that perhaps municipalities attempt to remedy service sectors that they find particularly challenging.

Box 1 **Commentary: Lived Experience by Solveig H. H. Kjus.**

I have personal experience of community and inpatient mental health services, both voluntary and compulsory hospitalisation. I commented on drafts of this article and contributed to discussions concerning the design of the project.

The study finds that higher levels of labour-years of GPs and mental health nurses were associated with lower levels of compulsory hospitalisation. This seems reasonable from a patient's view. The availability of GPs and mental health staff might secure and contribute to the alliance between the person and the healthcare system.

The study also indicates that more public housing was associated with lower levels of compulsory hospitalisation. Having a home that feels secure and comfortable is important for all people, also persons with SMI, and feeling secure and comfortable at home might reduce stress and deterioration that otherwise could end in a compulsory hospitalisation. The possibility to achieve this might be higher if the municipality has more public housing.

The study indicates that good cooperation between municipalities and specialist services was associated with lower levels of compulsory hospitalisation. This cooperation is important for the person to feel taken care of, and it increases the experience of continuity in the services, which is especially important for persons with SMI.

It is a limitation of the study that it does not include all involuntary *referrals*-only those that ended in a compulsory hospitalisation. The possible experience of being taken by force to compulsory hospitalisation is similar, even if the referral did not result in a compulsory hospitalisation. This might represent a trauma for the patient, next of kin, and other persons watching. A compulsory referral can therefore initiate that the person withdraws from future voluntary treatment, which, in turn, might end in new compulsory hospitalisations.

The geographical variation was larger for counts of hospitalisations than patients, and so were most associations. In combination, the variables on municipal mental health services could account for 5–6% of the total variation, which was equal to or less than the variation due solely to age distribution or area deprivation, and far less than the variation explained by the rates of individuals with SMI. This could indicate that improving municipal mental health services, at least the parameters included here, is no panacea for reducing the levels of compulsory hospitalisation. When all groups of variables were included in the same model, they were able to account for 39–40% of the variation according to the marginal *R*^2^. Further research is required to uncover other possible explanations for the geographical variation.

## Strengths and Limitations

The major strength of our study is that we had access to the entire population of people who were compulsorily hospitalised in Norway during the study period, yielding few selection problems. Furthermore, we employed a methodology that allowed us to separate variation at different levels of hierarchical clusters, as well as differentiating within- and between-area associations. Since we included data from all municipalities during the study period, these findings are likely to be representative of current practise.

The decision to also include measures of hospitalisations and not only patients, or first events, violate principles of independent observations that underlie the use of Poisson models and could result in deflated standard errors. This could be a concern if certain patients living in small municipalities contributed many hospitalisations; however, this was not a pervasive problem.

Since our study was exploratory, we did not adjust for multiple comparison (48). Future studies employing pre-planned hypotheses ought to be performed to confirm the associations observed in this study. Finally, the results from this study are not necessarily generalisable to other countries with different legislation and organisation of health services.

## Conclusion

This study shows considerable geographical variation in compulsory hospitalisation between municipalities. It indicates that increases in labour-years of GPs and mental health nurses, as well as public housing, are associated with lower levels of compulsory hospitalisation, as measured by inpatients and hospitalisations. Strengthening the municipal mental health services by providing resources for more GPs and mental health workers in the municipal services, and providing more public housing might thus contribute toward reaching health political ambitions of reducing the use of compulsory hospitalisation. This study also illustrates the importance of combining analyses of within- and between-area variation in longitudinal research on compulsory mental healthcare.

## Data Availability Statement

The data analysed in this study is subject to the following licences/restrictions: The data that support the findings of this study are available from the Norwegian Patient Registry and the Norwegian Directorate of Health. Restrictions apply to the availability of these data, which were used under licence for this study. Requests to access these datasets should be directed to https://www.helsedirektoratet.no/tema/statistikk-registre-og-rapporter/helsedata-og-helseregistre/norsk-pasientregister-npr/sok-om-data-fra-npr.

## Ethics Statement

Written informed consent for participation was not required for this study in accordance with the national legislation and the institutional requirements.

## Author Contributions

This study forms part of a larger research program for which JR, in collaboration with ON and TLH, obtained funding and accessed data. TH, JR, SOO, and TLH designed the present study. TH designed and performed the data analysis, created the figures, and wrote the first draft of the manuscript. SHHK wrote the lived experience commentary. All authors revised the manuscript in several rounds and approved the final version.

## Funding

The study is part of the ReCoN—Reducing Coercion in Norway research project, which was funded by the Research Council of Norway (project no. 273546).

## Conflict of Interest

The authors declare that the research was conducted in the absence of any commercial or financial relationships that could be construed as a potential conflict of interest.

## Publisher's Note

All claims expressed in this article are solely those of the authors and do not necessarily represent those of their affiliated organizations, or those of the publisher, the editors and the reviewers. Any product that may be evaluated in this article, or claim that may be made by its manufacturer, is not guaranteed or endorsed by the publisher.
