# Supplementary material for: Service Characteristics and Geographical Variation in Compulsory Hospitalisation: An Exploratory Random Effects Within–Between Analysis of Norwegian Municipalities, 2015–2018
Source: Front Psychiatry. 2021 Dec 9;12:737698. doi: 10.3389/fpsyt.2021.737698 (PMC8695843; doi:10.3389/fpsyt.2021.737698)
Supplement: Supplementary file 1 [file Data_Sheet_1.PDF]

Appendix A: Data sources

Individual level data on all contacts with specialist services in Norway are routinely recorded in the NPR. We acquired data for each episode of compulsory hospitalisation during 2015-2018. Counts were based on the patients' residency at the time of hospitalisation, and the number of compulsory hospitalisations were counted for each individual, within each municipality. Consequently, patients who moved during the study period were counted in multiple municipalities, but each episode was only counted once.

Information on population, public housing, and labour-years of GPs and mental health nurses was collected from Statistics Norway. Labour-years are defined as the number of full-time equivalent positions during a year. Information on the remaining service characteristics was obtained from a dataset on municipal mental health and addiction services (33). Unemployment data were provided by the Norwegian Labour and Welfare Administration.

For total number of labour-years in municipal mental health and addiction services, detailed information was unavailable for city districts in Bergen, Trondheim, and Stavanger. The municipal total was allotted according to each city districts' population under the assumption that labour-years were equally distributed across city districts. Labour-years of GPs included approximately 20% municipal employed physicians in other functions. For the number of mental health nurses, detailed information was lacking in all four cities, so the city total was allotted according to the distribution of total number of labour-years in mental health services.

Data from the Norwegian Patient Registry (NPR)

NPR contains information on all contacts with specialised services, and we received access to information on all episodes of compulsory hospitalisation and admissions converted to compulsory care during the study period, including age and gender of the patients, legal formality and dates of formality change, admission date, discharge date, institution. Furthermore, we received data on all contacts for people diagnosed with a severe mental disorder.

Data from Statistics Norway

Population, by sex and one-year age groups:
https://www.ssb.no/en/statbank/table/07459

Labour-years for physicians in the municipal health and care services:
https://www.ssb.no/en/statbank/table/11996 (municipalities)
https://www.ssb.no/en/statbank/table/12405 (urban districts)

Care services, labour-years, by education:
Registered nurses for the mentally subnormal (sic!)
https://www.ssb.no/en/statbank/table/11924

Public housing:
"A dwelling with municipal right of disposal is a dwelling that the municipality can rent to its inhabitants through a tenancy agreement, for example nursing and care dwellings, dwellings

for refugees, dwellings for socially and/or financially disadvantaged etc. Nursing homes, old people's homes etc without tenancy agreement are not included."
https://www.ssb.no/en/statbank/table/12008 (municipalities)
https://www.ssb.no/en/statbank/table/13203 (urban districts)

Crowded dwelling: "Lives in crowded dwelling, many rooms and sq.m.: Households are considered as living crowded if: 1. the number of rooms is lower than the number of residents or one resident lives in one room, and 2. the number of square metres (P-area) is below 25 sq.m. per person. If the number of rooms or the P-area is not specified, a household will be regarded as living in cramped conditions if one of these criteria is met."
https://www.ssb.no/en/statbank/table/11093

Data from the Norwegian Labour and Welfare Administration

Number of unemployed:
https://www.nav.no/no/nav-og-samfunn/statistikk/arbeidssokere-og-stillinger-statistikk/historisk-statistikk

Historical data from urban districts was graciously provided by email from the Norwegian Labour and Welfare Administration.

Data from IS 24/8 - Kommunalt psykisk helse- og rusarbeid

https://www.helsedirektoratet.no/rapporter/kommunalt-psykisk-helse-og-rusarbeid-%C3%A5rsrapporter_

IS 24/8 is a long-term research project commissioned by the national health authorities to follow the development of municipal mental health services since 1998. Data has been collected from every municipality since 2006. Since the mental health services are highly labour-intensive services, the resources allocated are measured in number of (full-time equivalent) person-labour years.

Total labour-years: How many labour-years are provided for people with mental and/ or addiction difficulties/illnesses? (This includes labour-years for physicians in the municipal health and care services)
Employment Support: Has the municipality used Individual Placement and Support (IPS)/Supported Employment (SE) in mental health work? (Yes/No).
User perspectives: Has the municipality in a systematic way gathered experiences from users of mental health and addiction work during the last 12 months, as basis for improving services? (Yes/No).
Recovery: To what degree would you say that mental health and addiction services in your municipality are recovery oriented? (To a very high degree, to a high degree, to some degree, to a small degree, to a very small degree).
Quality of cooperation: How do you evaluate that the cooperation agreement between municipality and health trust is working for adults with mental health difficulties/illness? (Very good/Good/Medium/Poor/Very poor)

Early Intervention: Has the municipality made efforts to uncover mental health or addiction problems as early as possible? (Yes/No).
Housing First: Has the municipality employed ”Housing First”? (Yes/No).

Appendix B: Methods

The hierarchical models were fit using equations of the following structure:

$$
\begin{aligned}
&\text{Hospitalisations/Patients}_i \sim Poisson(\lambda_i) \\
&\log(\lambda_i) = \log(population\ at\ risk) + \alpha_{j[i],k[i]} + \\
&\beta_1(year_{2016}) + \beta_2(year_{2017}) + \beta_3(year_{2018}) + \\
&\quad \beta_4(Population\ share\ aged\ 20\ to\ 39\ within) + \beta_5(Population\ share\ aged\ 20\ to\ 39\ between) + \\
&\quad \beta_6(Population\ share\ over\ 65\ within) + \beta_7(Population\ share\ over\ 65\ between) + \\
&\quad \beta_8(SMI\ per\ 1000\ within) + \beta_9(SMI\ per\ 1000\ between) + \\
&\quad \beta_{10}(Share\ in\ crowded\ dwelling\ within) + \beta_{11}(Share\ in\ crowded\ dwelling\ between) + \\
&\quad \beta_{12}(Unemployment\ share\ within) + \beta_{13}(Unemployment\ share\ between) + \\
&\quad \beta_{14}(Service\ characteristic\ within) + \beta_{15}(Service\ characteristic\ between), \\
&\alpha_j \sim N\left(\mu_{\alpha_j}, \sigma^2_{\alpha_j}\right), for\ municipality\ in\ CMHC\ catchment\ area\ j = 1, \ldots, J \\
&\alpha_k \sim N\left(\mu_{\alpha_k}, \sigma^2_{\alpha_k}\right), for\ CMHC\ catchment\ area\ k = 1, \ldots, K
\end{aligned}
$$

In order to quantify predicted change in the level of compulsory hospitalisation between and within-areas, conditional effect plots were created for the four continuous explanatory variables. Within-area associations were calculated at low, medium, and high average levels of the variable in question, where low values included areas within the first quartile, medium included areas between the first and third quartile, and areas above the third quartile were considered high.

Appendix C: Results

Unadjusted hierarchical models

Figure 1A shows exponentiated regression coefficients from eight different multilevel Poisson models which can be interpreted as rate ratios. They represent the relative change in the annual rate of patients or hospitalisations per population that would be expected for a one-unit increase in the explanatory variable. These models are not adjusted for control variables.

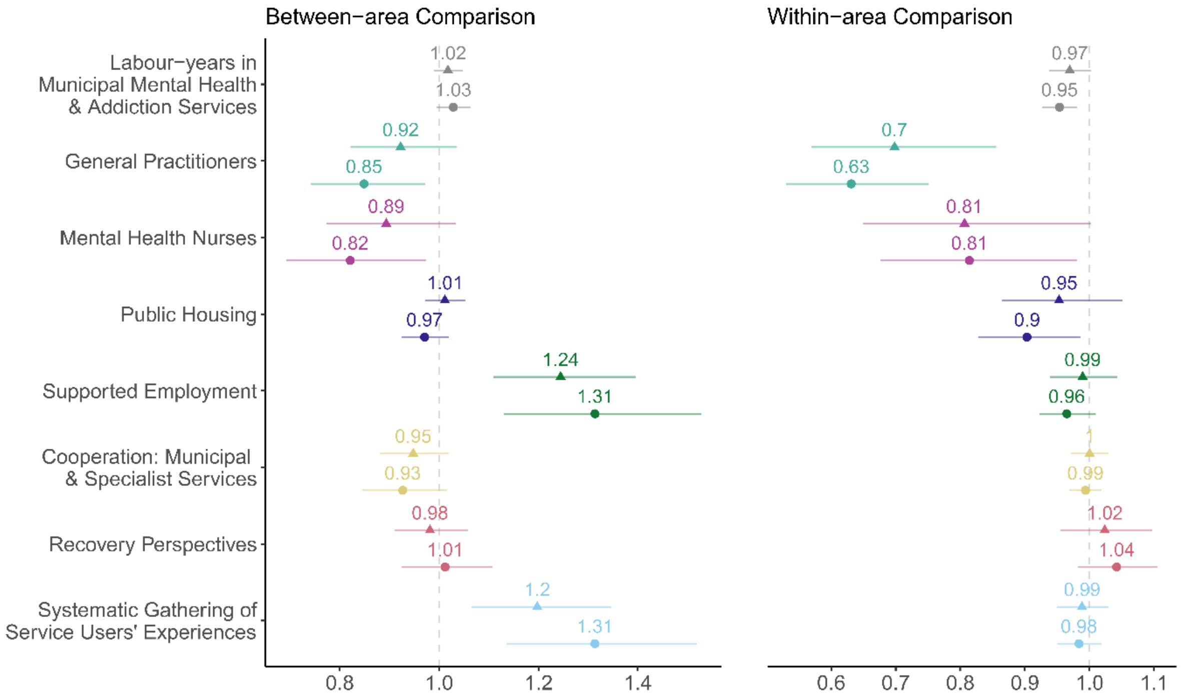

Figure 1A: Unadjusted between- and within-area associations of municipal mental health service in Norway 2015-2018. ▲ = Patients. ● = Hospitalisations. Rate ratios with 95% Wald confidence intervals.

Predicted values of compulsory hospitalisations and patients

Figure 2A shows the predicted values of compulsory hospitalisations and patients per 10 000 inhabitants associated with changes in municipal services, keeping control variables at their mean level. The right column of the figure shows the between-association, and the rug plot, which are the small lines above the x-axis, represents variation in the area means of the particular variable. The shaded ribbons represent 95% confidence intervals. The left column shows within-area associations at low, medium, and high average values of each variable in question. The x-axis and the accompanying rug plot show the distribution of deviations from the within-area average. The dashed lines represent the expected change for rates of compulsory hospitalised patients, while the solid lines show expected change in compulsory hospitalisation rates. The top row illustrates how the associations with total number of labour-years in municipal mental health per 1 000 differ, with a slightly negative within-effect and a positive aggregate cross-sectional association. The second and third row shows that the association of labour-years of general practitioners and mental health nurses is roughly the same within- and between-areas, but the within-effect is somewhat stronger for mental health nurses.

A 12-16% increase of general practitioners per population from the area average is associated with a reduction of one compulsory hospitalisation. The fourth row shows how within-area deviations in public housing per 100 have near zero effect on compulsory hospitalised patients, but a negative effect on compulsory hospitalisations, and a negative between-

association for both outcomes. A 42% increase of municipal residencies per population from the area average in areas with high average levels, and a 67% increase of municipal residencies in areas with low average levels is associated with a reduction of one compulsory hospitalisation.

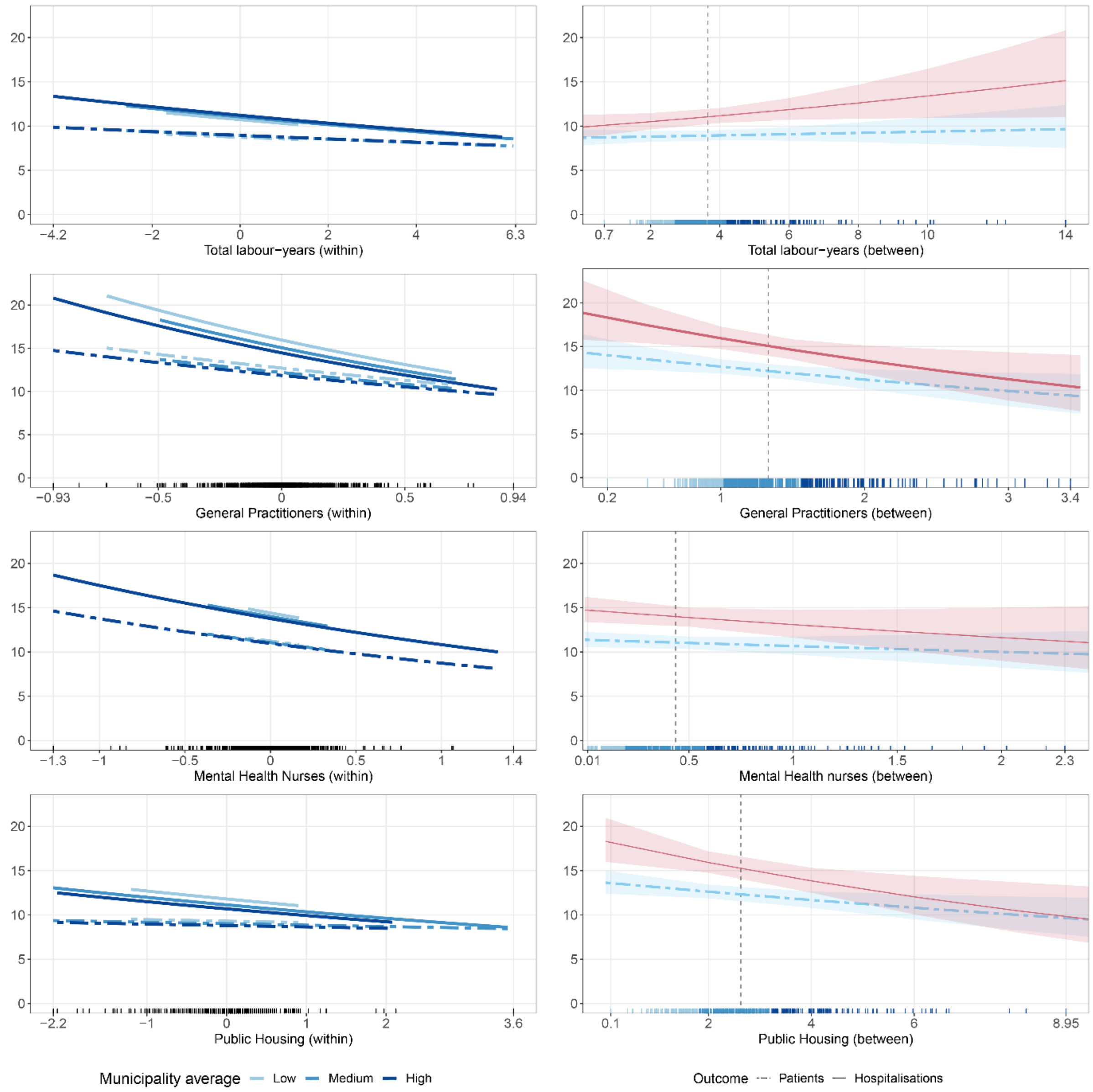

Figure 2A: Conditional effect plots, predicted average change in number of compulsory hospitalised patients and hospitalisations per 10 000 inhabitants within- and between municipalities in Norway 2015-2018. Y-axis shows predicted number of compulsory hospitalisations and compulsory hospitalised patients per 10 000, keeping control variables at their mean value, while the x-axis shows change in each explanatory variable per 1 000 inhabitant and includes minimum and maximum value. Dotted vertical line shows country average. Ribbons represent 95% confidence intervals.

1.4 Model performance and robustness checks

To test the appropriateness of the cluster structure, separate models were run using only municipality or CMHC catchment area as random intercept. Likelihood ratio tests showed that the models where municipalities were nested within CMHC catchment areas performed significantly better. One assumption of Poisson regression is that the variance is equal to the mean. Out of the final 32 estimated mixed models (eight unadjusted and eight fully adjusted, with patients and hospitalisations as outcomes), only one had significant overdispersion, with a dispersion parameter = 1.068. Nevertheless, we also calculated standard errors via quasi-likelihood by multiplying the regular standard errors with the square root of the dispersion parameter for all mixed models (1). The difference in standard errors had little impact on interpretation for any model. Furthermore, the inclusion of year as either fixed effect or random intercept had virtually no impact on coefficients, and the minor changes in standard errors did not affect interpretation.

Since some of the explanatory variables showed a slight increasing trend during the study period, estimation of the between-association using the area average could be biased by time. Following suggestions of Curran and Bauer (2) we de-trended the time-varying predictors by running area fixed effect linear regressions of the time-varying predictors on grand-mean centred time. The fitted values and residuals were then used to estimate the between- and within-effects. The resulting minor changes in coefficients and standard errors did not alter interpretation.

Not all within and between associations were significantly different, which could suggest that a standard random effects model would be more efficient for those explanatory variables (3). For ease of comparison, we decided to maintain the more general random effects within-between approach throughout.

Unlike the situation with linear models by ordinary least squares regression, the within-estimates obtained by within-between Poisson regression are similar, but not identical to those obtained using fixed effects. As another test of robustness, we therefore used the fixed effects Poisson estimator with log of population-at-risk as offset, and municipality and year as fixed effects, with and without the same set of control variables used in the main analysis. The resulting coefficients and standard errors were very similar to the within-estimates from the main analysis.

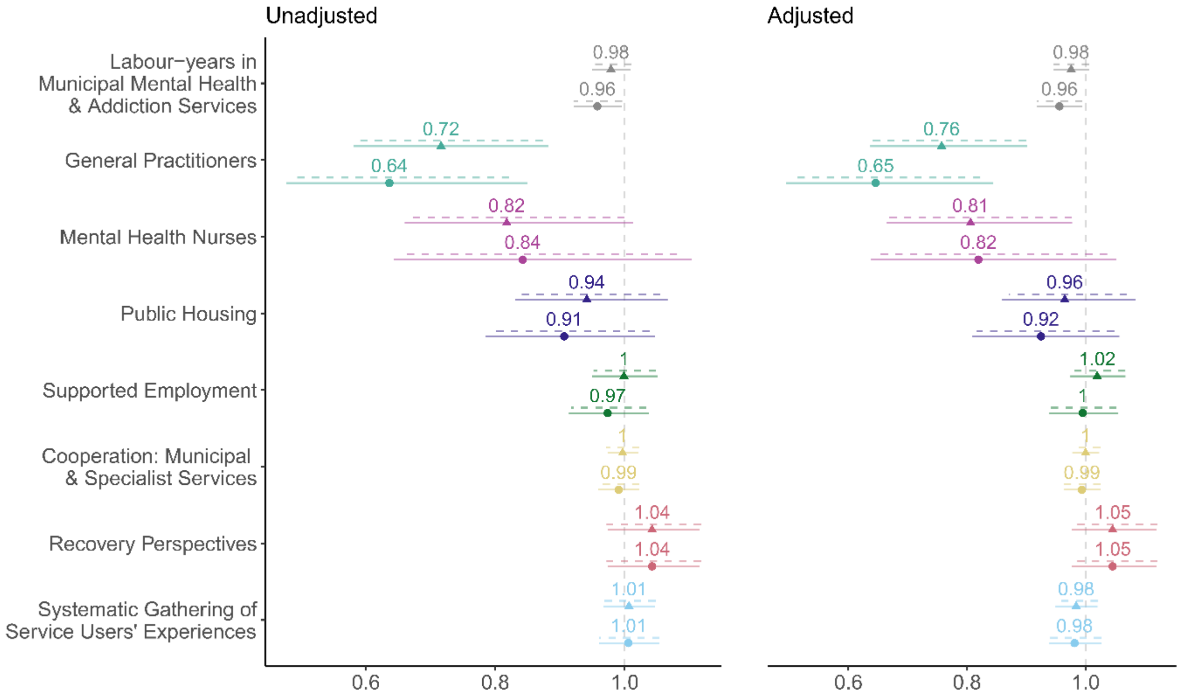

Figure 3A: Poisson estimator with municipality and year fixed effects. Solid lines show 95% confidence intervals based on municipality clustered standard errors, while dashed lines are based on White's heteroskedasticity robust SEs.

1. Gelman A, Hill J. Data analysis using regression and multilevel/hierarchical models. Cambridge university press; 2006.

2. Curran PJ, Bauer DJ. The Disaggregation of Within-Person and Between-Person Effects in Longitudinal Models of Change. Annu Rev Psychol. 2011;62(1):583–619.

3. Bell A, Fairbrother M, Jones K. Fixed and random effects models: making an informed choice. Qual Quant. 2019 Mar 1;53(2):1051–74.
